# Applying UTAUT2 model to investigate acceptance of DeepSeek by Chinese university students for academic use

**DOI:** 10.3389/fpsyg.2026.1828331

**Published:** 2026-05-15

**Authors:** Si Xu, Ge Zhang

**Affiliations:** College of Education, Binzhou Polytechnic, Binzhou, China

**Keywords:** behavioral intention, DeepSeek, university students, use behavior, UTAUT2

## Abstract

**Background:**

With generative AI increasingly embedded in higher education, understanding the factors that shape students' adoption of AI-driven learning assistants has become essential. Drawing on the UTAUT2 framework, this study investigates Chinese university students' acceptance and actual use of DeepSeek, an open-source general-purpose large language model.

**Methods:**

Survey data from 480 students were analyzed using confirmatory factor analysis and structural equation modeling.

**Results:**

The results indicate that performance expectancy, facilitating conditions, price value, and habit significantly predict behavioral intention, while behavioral intention, facilitating conditions, and habit predict actual usage. Habit emerged as the strongest determinant of both intention and use, highlighting the central role of routinization in sustaining engagement with educational AI.

**Conclusion:**

These findings highlight the importance of routinization in sustaining engagement with educational AI. The study extends the UTAUT2 framework to the context of large language models and offers practical implications for universities and developers in promoting both initial adoption and continued use.

## 1 Introduction

Amid the ongoing deep integration of AI into the educational domain, intelligent virtual assistants are playing a transformative role in reshaping higher education's learning environment (Alotaibi, 2024). By harnessing sophisticated technologies like natural language processing and machine learning, these systems enable robust capabilities for information retrieval and analysis, effectively surpassing the conventional constraints of relying solely on library holdings or static academic databases (Ofori-Boateng et al., 2024).

Through human-computer interaction, chatbots create deep interactive platforms, serving as key drivers of learning model transformation (Lv et al., 2022; Ye et al., 2024b). By breaking the constraints of time and space, they provide university students with tools for knowledge system development (Nicolescu and Tudorache, 2022). These technologies actively facilitate problem identification and solution-seeking, shifting learning from traditional passive reception to active exploration and deep thinking (Wang et al., 2024). This transformation aligns closely with constructivist learning theory, supporting students in constructing personalized and in-depth knowledge frameworks (Jayasinghe, 2024).

As an emerging AI-driven chat tool, DeepSeek has shown increasing potential in the field of higher education (Kerimbayev et al., 2024). Built on natural language processing and deep learning technologies, it not only excels in accurate information retrieval and natural language generation but also continuously refines response quality through interactive learning, thereby enhancing both language comprehension and coherence (Peng et al., 2025). In terms of learning support, DeepSeek offers a range of features including intelligent conversation, text generation, semantic understanding, and computational reasoning, all of which assist university students in efficiently accessing academic resources, refining their writing, and supporting logical reasoning and code development (Okaiyeto et al., 2025). Moreover, its open-source large reasoning model, DeepSeek-R1, is equipped with robust computational power (Poo, 2025), enabling it to perform online searches, engage in deep thinking modes, and process tasks such as file uploads, text scanning, and image recognition, thus providing students with a more comprehensive set of learning tools.

The swift progress of AI has sparked growing scholarly interest in the use of intelligent chat tools within educational research (Alqahtani et al., 2023). However, research on DeepSeek remains relatively scarce. While much of the existing literature centers on the educational applications of LLMs, notably exploring ChatGPT's effects on language learning, academic writing, and coding instruction (Subhani et al., 2025; Zhang et al., 2025), research specifically targeting DeepSeek, a newer AI-driven chat tool, remains in its early stages of development (Zhai et al., 2021). Preliminary findings indicate that DeepSeek demonstrates strong capabilities in text generation, reasoning, and knowledge retrieval, making it particularly valuable for academic writing support, problem-solving, and code generation (Gao et al., 2025). Furthermore, its open-source nature provides greater customizability and scalability compared to some proprietary AI tools, offering educators and students a more flexible and interactive learning experience in higher education.

Despite a growing body of research on general-purpose LLMs such as ChatGPT (Ye et al., 2025), there is limited empirical evidence specifically on DeepSeek. DeepSeek's open-source nature, customizable educational plugins, and enhanced support for academic file uploads may create different adoption dynamics compared with proprietary LLMs. This study therefore fills this gap by applying UTAUT2 to examine the determinants of Chinese students' intention to use and actual use of DeepSeek, contributing to theory by identifying boundary conditions for UTAUT2 in the context of domain-specific LLMs and providing practical guidance for educators and developers.

## 2 Theoretical framework

To examine Chinese university students' acceptance and usage of DeepSeek, this study adopts the UTAUT2 model as its theoretical foundation. UTAUT2 extends the original UTAUT by retaining Performance Expectancy (PE), Effort Expectancy (EE), Social Influence (SI), and Facilitating Conditions (FC), while incorporating Hedonic Motivation (HM), Price Value (PV), and Habit (HT). These additional constructs enhance explanatory power in consumer-oriented contexts (Venkatesh et al., 2012). Building on this, UTAUT2 provides a useful framework for examining students' adoption of AI-driven learning tools.

A growing body of research has demonstrated the applicability of the UTAUT2 model in explaining technology adoption in educational contexts, including e-learning, mobile learning, and, information and communication technology, more recently, large language models such as ChatGPT (Dahri et al., 2024; Grassini et al., 2024; Liu et al., 2025; Shahzad et al., 2025). Prior studies have shown that PE, EE, HM, and SI are important predictors of students' BI (Patil and Undale, 2023; Twum et al., 2021), while FC and HT tend to influence both BI and UB.

Despite these contributions, several gaps remain. Much of the existing research has concentrated on general-purpose AI tools, with comparatively limited attention given to emerging platforms such as DeepSeek. In addition, findings related to key variables, particularly EE, SI, and HM, have been somewhat inconsistent across different technological settings. Moreover, most studies focus primarily on behavioral intention, whereas fewer examine intention and actual usage together.

Against this backdrop, DeepSeek presents a distinctive case due to its open-source design, its orientation toward academic use, and its increasing adoption in the Chinese context. Building on these considerations, this study employs the UTAUT2 framework to investigate both behavioral intention and actual use, aiming to provide a more context-specific perspective on AI adoption in higher education.

### 2.1 Performance expectancy (PE)

PE is the belief that using a technology improves task performance (Venkatesh et al., 2012, 2003). In AI-supported learning environments, this construct extends beyond efficiency gains to include cognitive augmentation. Tools such as DeepSeek can assist students in information retrieval, idea generation, and problem-solving processes, thereby reshaping how academic tasks are completed. When students perceive that such AI functions meaningfully enhance their learning effectiveness or reduce cognitive burden, they are more likely to form favorable usage intentions.

H1: PE significantly positively affects university students' BI to use DeepSeek.

### 2.2 Effort expectancy (EE)

EE refers to the perceived ease of learning and using a technology (Venkatesh et al., 2012, 2003). Recent studies have emphasized its key role in university students' acceptance of artificial intelligence (Acosta-Enriquez et al., 2024). In the context of AI-based tools, perceived ease of use is closely tied to the transparency of interaction and the extent to which users can intuitively engage with complex functionalities. Although systems like DeepSeek integrate advanced capabilities such as multimodal reasoning, their adoption depends on whether users feel confident navigating these features without excessive effort. A lower perceived learning barrier may reduce uncertainty and encourage trial use, thereby strengthening behavioral intention.

H2: EE significantly positively affects university students' BI to use DeepSeek.

### 2.3 Social influence (SI)

SI captures the extent to which peers, instructors, or family shape one's decision to adopt a tool (Venkatesh et al., 2012, 2003). Previous studies have verified the significant impact of SI on various technological applications, such as blended learning (Rudhumbu, 2022), social networks (Gharrah et al., 2021), and AI-enabled (Liu et al., 2025). For DeepSeek, this includes whether encouragement or concerns from academic communities affect students' intentions.

H3: SI significantly positively affects university students' BI to use DeepSeek.

### 2.4 Facilitating conditions (FC)

FC refers to the availability of resources and support needed to use a technology (Venkatesh et al., 2012, 2003). FC has been proven to be a significant factor influencing technology adoption, such as in online learning (Goto and Munyai, 2022) and virtual reality (VR) (Al Farsi, 2023). Within AI-mediated learning contexts, FC refer to the availability of technical infrastructure, institutional support, and guidance that enable effective use of AI tools.

H4: FC significantly positively affects university students' BI to use DeepSeek.

### 2.5 Hedonic motivation (HM)

HM is the enjoyment derived from using a technology (Venkatesh et al., 2012, 2003). Previous studies have confirmed the importance of HM in technological contexts, such as open online courses (Amid and Din, 2021; Ye et al., 2022), MOOCs (Meet et al., 2022), and mobile learning (García de Blanes Sebastián et al., 2025). Unlike entertainment-oriented systems, AI learning tools are primarily task-driven. However, interactive features such as conversational feedback and adaptive responses may still generate a sense of engagement. This experiential aspect can enhance users' overall perception of the tool, even when their primary goal remains academic. Therefore, enjoyment may act as a complementary rather than dominant driver of intention.

H5: HM significantly positively affects university students' BI to use DeepSeek.

### 2.6 Price value (PV)

PV is the trade-off between benefits and costs of technology use (Venkatesh et al., 2012, 2003). Prior studies have indicated that PV is a key factor influencing university students' willingness to adopt tools like ChatGPT (Sinaga et al., 2024), blended learning (Rudhumbu, 2022) and online learning platforms (Gharaibeh, 2023). In AI learning scenarios, cost is not limited to monetary expenditure but also includes time investment and cognitive effort required to learn the system. Students are more inclined to adopt AI tools when the perceived academic advantages outweigh these costs.

H6: PV significantly positively affects university students' BI to use DeepSeek.

### 2.7 Habit (HT)

HT refers to the automaticity of technology use formed through repeated prior experience (Venkatesh et al., 2012, 2003). Existing research has highlighted HT as a significant predictor of students‘ intention to use various technologies, including social media platforms (Gharrah et al., 2021), smartphones (Nikolopoulou et al., 2020), and AI tools like ChatGPT (GC et al., 2024). In AI-supported learning environments, repeated interaction with tools like DeepSeek can gradually embed them into students' study routines. As usage becomes less deliberate and more habitual, reliance on the tool increases, strengthening intention even without conscious evaluation.

H7: HT significantly positively affects university students' BI to use DeepSeek.

### 2.8 Behavioral intention(BI)

BI is an individual's willingness to use a technology (Venkatesh et al., 2012, 2003). In AI-based academic settings, intention captures the extent to which students are willing to integrate tools like DeepSeek into their learning practices. A stronger intention is generally associated with a higher likelihood of sustained use.

H8: BI significantly positively affects university students' UB of DeepSeek.

### 2.9 Use behavior (UB)

UB describes the actual extent of technology use (Venkatesh et al., 2012, 2003). In the context of university students using ChatGPT (Grassini et al., 2024), blended learning (Rudhumbu, 2022), and e-learning (García de Blanes Sebastián et al., 2025), BI and UB are crucial indicators of technology acceptance and continued usage. In the present study, UB is characterized by how often and to what degree university students incorporate DeepSeek into their academic activities. Existing studies have confirmed that FC directly influence university students' behavior in using e-learning platforms (Zacharis and Nikolopoulou, 2022) and Google Classroom (Celedonio and Picaso, 2023).

H9: FC significantly positively affects university students' UB of DeepSeek.

Meanwhile, another important factor—HT—significantly influences university students' UB in mobile learning (García de Blanes Sebastián et al., 2025), technology adoption (Bessadok and Hersi, 2023), and Google Classroom (Celedonio and Picaso, 2023). Therefore, Hypothesis 10 is proposed:

H10: HT significantly positively affects university students' UB of DeepSeek.

## 3 Method

This study conducted a questionnaire survey among Chinese university students. To begin with, CFA was conducted to assess the reliability and validity of the measurement model. Subsequently, SEM was utilized to analyze the hypothesized path relationships among constructs, with the goal of uncovering the primary factors driving Chinese university students' adoption of DeepSeek.

### 3.1 Participants and procedure

Data were collected between March 10 and April 10, 2025 through an online survey administered on the wjx platform (similar to SurveyMonkey). Participants were recruited via course-related channels and student social networks, allowing responses to be obtained from multiple universities. This approach relied on accessibility rather than random selection and can therefore be classified as convenience sampling. As a result, the sample may be biased toward students who are more active in online environments or more familiar with AI tools.

All participants were informed of the study's purpose prior to completing the questionnaire and were assured of anonymity and voluntary participation. To improve data quality, responses with extremely short completion times or obvious response patterns were removed. After screening, 480 valid responses were retained for analysis.

Table 1 provides a detailed overview of the study sample, which includes 186 male and 294 female participants. The age distribution of the university students in the sample is as follows: 165 participants were under 19, 247 were aged 20 to 22, 47 were between 23 and 25, and 21 were over 26.

**Table 1:** Demographic information of participants (*N* = 480).

Gender	*n*	%	Age	*N*	%
Male	186	38.750	≤ 19	165	34.375
Female	294	61.250	20–22	247	51.458
	23–25	47	9.792
	≥26	21	4.375

This table presents an overview of the study sample based on gender and age.

### 3.2 Measures

The UTAUT2 model's ability to explain and predict technology adoption behavior in education has been extensively validated (Sergeeva et al., 2025; Sinaga et al., 2024; Talan et al., 2024). The measurement items were adapted from validated UTAUT2 scales (Venkatesh et al., 2012) and tailored to the context of DeepSeek. Each construct was operationalized to capture its relevance to this tool.

To ensure linguistic and cultural equivalence, the items were translated into Chinese following a forward–back translation procedure. Two bilingual experts independently produced forward translations, discrepancies were reconciled, and an independent translator conducted back-translation. A pilot test with 30 students confirmed clarity and appropriateness, leading to minor revisions.

### 3.3 Data analysis

This study utilized SPSS and AMOS software for data analysis, following a two-stage process to ensure data reliability and validity while testing the research hypotheses.

#### 3.3.1 Stage 1: Measurement Model Evaluation

Descriptive statistics were first computed to profile the sample. Reliability was assessed through Cronbach's alpha, while construct validity was examined using CR (Churchill, 1979) and AVE (Farrell, 2010). Discriminant validity was examined using both the Fornell-Larcker criterion (Fornell and Larcker, 1981) and the heterotrait-monotrait (HTMT) ratio (Henseler et al., 2015). To address potential common method bias, Harman's single-factor test and a CFA-based common latent factor (CLF) approach were employed (Podsakoff et al., 2003).

#### 3.3.2 Stage 2: Structural Model Testing

After validating the measurement model, SEM was employed to test the hypothesized relationships among constructs within the UTAUT2 framework. Path coefficients and model fit indices were used to assess the explanatory power and predictive accuracy of the model in explaining Chinese university students' acceptance and actual usage of DeepSeek.

## 4 Results

### 4.1 Measurement model

The UTAUT2 questionnaire demonstrated robust measurement quality with strong reliability and validity. As shown in Table 2, Cronbach's alpha values for the constructs ranged from.922 to.949, with an overall value of.981, all exceeding the recommended threshold of 0.700 (Hair et al., 2010). CR values ranged from 0.922 to 0.950, surpassing the 0.700 criterion and confirming scale reliability (Churchill, 1979), AVE values ranged from 0.746 to 0.864, all exceeding the recommended cutoff of 0.500 (Farrell, 2010), providing strong evidence of convergent validity. Moreover, all factor loadings ranged from 0.823 to 0.937, substantially higher than the 0.400 (Guadagnoli and Velicer, 1988), showing that the items strongly represented their respective constructs.

**Table 2:** Descriptive statistics, and reliability and convergent validity measures.

Constructs	Mean	SD	No. of items	Cronbach's alpha	Factor loading	CR	AVE
PE	5.584	1.150	4	0.935	0.830–0.937	0.936	0.786
EE	5.498	1.164	4	0.922	0.823–0.914	0.925	0.755
SI	5.226	1.282	3	0.935	0.898–0.918	0.935	0.828
FC	5.432	1.127	4	0.922	0.852–0.886	0.922	0.746
HM	5.340	1.257	3	0.928	0.876–0.930	0.931	0.818
PV	5.172	1.327	3	0.949	0.914–0.937	0.950	0.864
HT	4.554	1.529	4	0.937	0.859–0.913	0.939	0.794
BI	5.195	1.298	3	0.940	0.899–0.931	0.941	0.842
UB	5.076	1.272	4	0.925	0.805–0.917	0.929	0.766

Descriptive statistics further indicated that the mean scores for PE (M = 5.584, SD = 1.150), EE (M = 5.498, SD = 1.164), SI (M = 5.226, SD = 1.282), FC (M = 5.432, SD = 1.127), HM (M = 5.340, SD = 1.257), PV (M = 5.172, SD = 1.327), BI (M = 5.195, SD = 1.298), and UB (M = 5.076, SD = 1.272) were all above the midpoint of 4 on the 7-point Likert scale. This suggests a generally moderate-to-high level of agreement among respondents, with PE receiving the highest rating, reflecting students' recognition of DeepSeek's practical value, while HT had the lowest score, implying that its use had not yet become habitual.

To assess potential multicollinearity among predictors, VIF and Tolerance values were computed separately for the models predicting BI and UB (Table 3). For the BI model, VIF values ranged from 2.211 to 5.958 and tolerance values ranged from 0.168 to 0.452. For the UB model, VIF values ranged from 2.177 to 3.244 and tolerance values from 0.308 to 0.459.

**Table 3:** Multicollinearity diagnostics for BI and UB models.

Model	Variable	VIF	Tolerance
BI	PEM	3.497	0.286
BI	EEM	4.598	0.217
BI	SIM	3.957	0.253
BI	FCM	5.958	0.168
BI	HMM	5.074	0.197
BI	PVM	3.442	0.291
BI	HTM	2.211	0.452
UB	FCM	2.177	0.459
UB	HTM	2.340	0.427
UB	BIM	3.244	0.308

Although some VIF values slightly exceed the more conservative threshold of 5.0, they remain below the commonly accepted upper limit of 10.0 (Hair et al., 2019). It has also been noted that VIF thresholds for detecting multicollinearity are often reported within a range of 5 to 10, depending on the research context (Kim, 2019). Moreover, prior research cautions against rigid reliance on such cutoff values and emphasizes that multicollinearity should be evaluated in relation to model structure and study context rather than based solely on fixed thresholds (O'brien, 2007).

### 4.2 Structural model

To assess the discriminant validity between latent variables, this study used the method outlined by Fornell and Larcker (1981). As shown in Table 4, the square roots of the AVE values (diagonal) exceeded the corresponding inter-construct correlations (off-diagonal), confirming adequate discriminant validity. Although the correlations among EE, FC, and HM were relatively high (above 0.800), their AVE square roots remained greater than these correlations. This suggests that each construct shares more variance with its own indicators than with other constructs, supporting their empirical distinctiveness.

**Table 4:** Discriminant validity matrix.

Constructs	PE	EE	SI	FC	HM	PV	HT	BI	UB
PE	**0.887**								
EE	0.814^**^	**0.869**							
SI	0.728^**^	0.744^**^	**0.910**						
FC	0.774^**^	0.841^**^	0.819^**^	**0.864**					
HM	0.768^**^	0.800^**^	0.787^**^	0.860^**^	**0.905**				
PV	0.693^**^	0.734^**^	0.724^**^	0.760^**^	0.790^**^	**0.929**			
HT	0.528^**^	0.569^**^	0.682^**^	0.597^**^	0.620^**^	0.686^**^	**0.891**		
BI	0.706^**^	0.687^**^	0.703^**^	0.732^**^	0.739^**^	0.759^**^	0.754^**^	**0.918**	
UB	0.657^**^	0.657^**^	0.706^**^	0.699^**^	0.734^**^	0.720^**^	0.800^**^	0.871^**^	**0.875**

The bold diagonal values represent the square root of the Average Variance Extracted (AVE). Off-diagonal values represent inter-construct correlations. ^**^p < 0.01.

In addition to the Fornell-Larcker criterion, the HTMT was employed to provide a more rigorous assessment of discriminant validity. As presented in Table 5, most HTMT values were assessed using the recommended threshold of 0.900 (Henseler et al., 2015), indicating satisfactory discriminant validity among the constructs. However, three construct pairs (EE-FC, FC-HM, and BI-UB) slightly exceeded the 0.900 threshold, indicating some conceptual proximity among these constructs.

**Table 5:** HTMT ratio of correlations.

Constructs	PE	EE	SI	FC	HM	PV	HT	BI
EE	0.876	–						
SI	0.778	0.800	–					
FC	0.833	0.911	0.881	–				
HM	0.824	0.864	0.843	0.930	–			
PV	0.735	0.784	0.768	0.812	0.839	–		
HT	0.571	0.619	0.732	0.649	0.670	0.733	–	
BI	0.753	0.738	0.750	0.786	0.791	0.803	0.807	–
UB	0.708	0.711	0.757	0.757	0.792	0.768	0.859	0.933

Given that the Fornell-Larcker criterion was satisfied and all constructs demonstrated adequate reliability and convergent validity, these results indicate acceptable but not perfect discriminant validity. This pattern is theoretically plausible within the UTAUT2 framework, where several constructs capture closely related aspects of users' perceptions and behavioral processes (Venkatesh et al., 2012).

To evaluate model fit, multiple indices were examined against commonly recommended thresholds (Table 6). The CMIN/DF value is 4.071, which is below the suggested upper limit of 5.0, indicating an acceptable level of fit (Schumacker and Lomax, 2004). The GFI value of 0.811 exceeds the minimum recommended threshold of 0.800, suggesting an adequate, though not particularly strong, model fit (Byrne, 2013). The RMSEA is.080, which lies at the upper boundary of the acceptable range, indicating a reasonable but marginal fit (McDonald and Ho, 2002). In addition, the SRMR value of 0.049 is well below the recommended threshold of 0.080, further supporting a good model fit (Hu and Bentler, 1999). In terms of incremental fit, the CFI (0.930), NFI (0.910), TLI (0.920), and IFI (0.930) all exceed the recommended threshold of 0.900, suggesting satisfactory model fit (Shek and Yu, 2014). For parsimonious fit, PNFI (0.794), PCFI (0.812), and PGFI (0.665) are all above the suggested minimum of 0.500, indicating an acceptable balance between model complexity and fit (Byrne, 2013). Overall, the model demonstrates an acceptable level of fit according to commonly used SEM criteria, although some indices suggest that there remains room for further improvement.

**Table 6:** Goodness of fit measure.

Overall fit	Index	Values obtained	Criteria	Fit indices
Absolute fit	CMIN/DF	4.071	<5	Acceptable (Schumacker and Lomax, 2004)
	GFI	0.811	≥0.800	Acceptable (Byrne, 2013)
	RMSEA	0.080	≤ 0.080	Acceptable (McDonald and Ho, 2002)
	SRMR	0.049	≤ 0.080	Acceptable (Hu and Bentler, 1999)
Incremental fit	CFI	0.930	≥0.900	Acceptable (Shek and Yu, 2014)
	NFI	0.910	≥0.900	
	TLI	0.920	≥0.900	
	IFI	0.930	≥0.900	
Parsimonious fit	PNFI	0.794	≥0.500	Acceptable (Byrne, 2013)
	PCFI	0.812	≥0.500	
	PGFI	0.665	≥0.500	

Given that the data were collected using a self-reported questionnaire, the potential influence of common method bias (CMB) was further assessed. In addition to procedural remedies such as anonymity and voluntary participation, a CLF was introduced into the measurement model (Podsakoff et al., 2003). All observed items were allowed to load on both their respective constructs and the latent method factor. The comparison between the original model and the CLF model indicated an improvement in model fit, with CFI increasing from 0.930 to 0.951 and TLI from 0.920 to 0.940. The differences in standardized factor loadings between the original model and the CLF model were generally below 0.200, suggesting that CMB is unlikely to bias the results substantially.

The results of the path analysis (Table 7) show that PE (β = 0.319, *p* < 0.001), FC (β = 0.081, *p* < 0.05), PV (β = 0.172, *p* < 0.05), and HT (β = 0.483, *p* < 0.001) have significant positive effects on BI, thus supporting hypotheses H1, H4, H6, and H7. Among these, HT exerts the strongest effect, indicating its dominant role in shaping behavioral intention. In contrast, EE (β = −0.184, *p* < 0.05), SI (β = −0.192, *p* < 0.05), and HM (β = 0.090, *p* >0.05) do not significantly affect BI, leading to the rejection of H2, H3, and H5. This divergence from prior studies may reflect contextual differences in students' adoption of AI tools, suggesting that ease of use, social influence, and hedonic motivation are less decisive when evaluating a novel academic application such as DeepSeek. Additionally, BI (β = 0.689, *p* < 0.001) has a significant positive effect on UB, supporting H8. FC (β = 0.081, *p* < 0.05) and HT (β = 0.230, *p* < 0.001) also positively influence UB, supporting H9 and H10. Among these, BI shows the strongest effect, highlighting its central role in explaining actual UB.

**Table 7:** Hypothesis testing results.

Hypothesis	Relationship	Results	Conclusion
H1	PE → BI	0.319^***^	Supported
H2	EE → BI	−0.184^*^	Not Supported
H3	SI → BI	−0.192^*^	Not Supported
H4	FC → BI	0.081^*^	Supported
H5	HM → BI	0.090	Not Supported
H6	PV → BI	0.172^*^	Supported
H7	HT → BI	0.483^***^	Supported
H8	BI → UB	0.689^***^	Supported
H9	FC → UB	0.081^*^	Supported
H10	HT → UB	0.230^***^	Supported

This table presents a summary of the research hypothesis results. ^*^p < 0.05, ^**^p < 0.01, ^***^p < 0.001.

**Figure 1:**
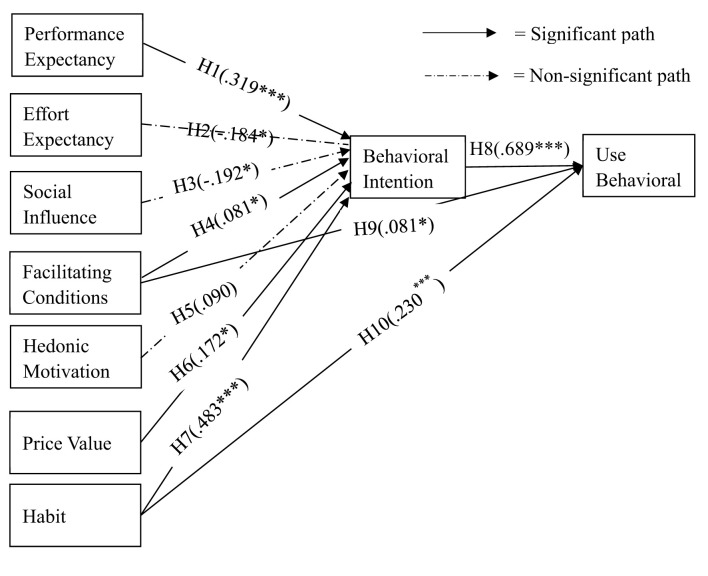
Structural measurement model. The figure illustrates the relationships between the variables, the proposed path coefficients, and the outcomes. ^*^p < 0.05, ^**^p < 0.01, ^***^p < 0.001.

Overall, seven hypotheses (H1, H4, H6, H7, H8, H9, and H10) were supported, while three (H2, H3, and H5) were not. Figure 1 visually presents the structural model with standardized path coefficients, illustrating both the significant and non-significant relationships identified in the analysis.

## 5 Discussion

This study, grounded in the UTAUT2 model, examined Chinese university students' adoption of DeepSeek, an emerging AI tool in higher education. With AI technologies rapidly transforming learning environments (Alam and Mohanty, 2023), clarifying the mechanisms of technology acceptance holds both theoretical and practical importance (Lin et al., 2023). The findings highlight not only the drivers of behavioral intention and use behavior but also boundary conditions that may refine the applicability of UTAUT2 in novel educational contexts.

The supported hypotheses highlight the importance of PE, FC, PV, and HT in shaping students' adoption of DeepSeek. PE significantly predicted behavioral intention, underscoring students' perception that DeepSeek enhances learning efficiency and task completion. This is consistent with both the original UTAUT2 propositions (Venkatesh et al., 2012, 2003) and recent studies on ChatGPT adoption (Grassini et al., 2024), reinforcing that perceived utility remains a primary determinant of AI adoption in education.

FC emerged as a robust predictor of both BI and UB. This dual effect suggests that adequate technical infrastructure, institutional support, and accessible guidance are indispensable for sustained engagement. While previous research emphasizes functional advantages of educational technologies (Timotheou et al., 2023), the present findings indicate that without reliable support systems, the integration of AI tools like DeepSeek may falter despite their perceived benefits.

The significance of PV confirms that students weigh the cost–benefit balance when adopting new technologies. DeepSeek's current low-cost or free features have likely enhanced its acceptance, in line with prior evidence that favorable value perceptions foster adoption (Rudhumbu, 2022; Twum et al., 2021). Nevertheless, whether this acceptance persists under potential future monetization remains an open empirical question, warranting longitudinal monitoring of user attitudes.

The most striking result concerns HT, which showed the strongest effect on both BI and UB. This highlights that repeated positive experiences with DeepSeek can lead to routinized usage, echoing Gharrah et al. (2021). Practically, this finding suggests that institutions should not only promote initial uptake but also design interventions that encourage habit formation by embedding DeepSeek into regular coursework, incentivizing repeated use, and integrating it with existing learning platforms.

In contrast, EE and SI show significant negative relationships with BI, whereas HM does not reach statistical significance, which partly differs from earlier findings. This pattern suggests that the explanatory power of UTAUT2 may vary across AI-supported learning contexts such as DeepSeek. The negative effect of EE may be interpreted from multiple perspectives. First, university students, as digitally experienced users, may exhibit a certain degree of overconfidence when interacting with new technologies, which reduces their sensitivity to ease of use (Bag et al., 2020). Second, the use of AI tools often involves more complex cognitive processes, such as evaluating outputs and integrating information, which may increase cognitive load and shift attention away from usability toward task performance (Puppala, 2025). In this sense, ease of use may no longer function as a motivating factor and may even be perceived as less relevant in demanding learning scenarios.

A similar explanation applies to SI. The negative relationship suggests that students' adoption of AI tools is largely driven by individual judgment rather than social expectations. In self-directed learning environments, external influence from peers or instructors may be less influential, and in some cases, may even be perceived as constraining autonomy (Bolívar-Cruz and Verano-Tacoronte, 2025). By comparison, the non-significant result for HM indicates that students primarily treat DeepSeek as a functional resource rather than a source of enjoyment, which is consistent with Zhao et al. (2024). Overall, these findings suggest that in AI-based learning environments, factors related to autonomy, cognitive engagement, and task orientation may play a more central role than traditional determinants such as ease of use, social norms, and enjoyment.

## 6 Conclusion

Guided by the UTAUT2 model, this study conducted an empirical survey of 480 university students across 18 provinces in China. The findings reveal that PE, FC, PV, and HT significantly and positively predicted students' BI, while BI, FC, and HT further exerted significant positive effects on UB. Notably, HT emerged as the strongest predictor of both BI and UB, indicating that DeepSeek has gradually become embedded in students' daily academic routines and serves as an integral component of their intelligent learning environments.

Beyond confirming the robustness of UTAUT2, these findings contribute to theory by identifying potential boundary conditions of the model in DeepSeek contexts. While PE, FC, PV, and HT remain central drivers, the non-significant effects of EE, SI, and HM imply that factors typically important in other settings may lose salience when students adopt highly task-oriented, utility-driven AI tools. This nuance enriches the theoretical understanding of technology acceptance by highlighting the contextual contingencies of UTAUT2.

From a practical perspective, the study underscores the need for universities to go beyond tool performance optimization and actively cultivate the conditions that sustain adoption. This includes developing technical support systems, integrating AI tools into regular coursework, providing training to ensure positive initial experiences, and maintaining affordable access. Such measures not only promote initial uptake but also facilitate habit formation, which, as shown in this study, is essential for long-term engagement. In a broader sense, the findings suggest that fostering habitual use of AI-powered tools could be a cornerstone strategy for advancing intelligent learning environments and preparing students for continuous technological transformation in higher education (Liu et al., 2026).

## 7 Implications

### 7.1 Theoretical contributions

This study adds to the growing body of research on technology acceptance and AI-supported learning in higher education. It extends the UTAUT2 framework to the context of generative AI tools, an area that remains relatively underexplored. By examining students' use of DeepSeek, the study provides empirical insight into how established acceptance mechanisms function within an emerging AI-based learning environment.

The findings also offer a more context-sensitive understanding of the determinants within UTAUT2. While PE, FC, PV, and HT were found to significantly influence BI, EE, SI, and HM did not show significant effects. This pattern suggests that, in AI-supported learning contexts, students are more concerned with functional value and usage conditions than with ease of use or social influence. In addition, the results highlight the role of HT as a key driver of both BI and UB. Repeated interaction with AI tools appears to foster stable usage patterns, which in turn facilitate the transition from intention to behavior. Finally, by examining both BI and UB within a unified model, this study provides further insight into the linkage between intention formation and actual use. The findings indicate that FC and HT play an important role in this process, shedding light on how AI tools become embedded in students' everyday learning practices.

### 7.2 Practical implications

The findings provide several practical implications for stakeholders involved in AI-supported learning environments. For educators, it is important to strengthen students' perceptions of usefulness and provide structured guidance in using AI tools. Integrating AI-based tasks into course activities and offering timely support can help students better understand the value of such tools. For educational institutions, the significant role of facilitating conditions highlights the need for adequate technical and organizational support. Universities should improve digital infrastructure, provide training on AI-assisted learning, and establish clear guidelines for responsible use (Ye et al., 2024a). For students, the strong effect of habit suggests that consistent engagement with AI tools is essential. Students are encouraged to develop regular usage patterns by incorporating AI tools into their daily study routines, while maintaining critical thinking when engaging with AI-generated content.

## 8 Limitations and recommendations

This study offers an initial insight into how university students engage with DeepSeek; however, several limitations should be acknowledged. First, the use of convenience sampling may have influenced the composition of the sample. As participation relied on online access and voluntary responses, students who are more familiar with digital technologies or already interested in AI may have been more inclined to participate. This potential self-selection bias may limit the generalizability of the findings.

Second, the study does not explicitly control for demographic or contextual variables such as gender, age, academic discipline, institutional type, regional background, or prior experience with AI tools. Although the sample is relatively homogeneous, consisting mainly of university students with similar educational backgrounds and levels of digital exposure, these factors may still shape students' access to AI technologies, as well as their perceptions and usage patterns. As a result, the findings should be interpreted with caution when applied to broader populations. Future research could incorporate these variables to examine potential differences across subgroups and improve the robustness of the results.

Third, the cross-sectional design limits the ability to capture changes in students' perceptions and behaviors over time. Longitudinal or experimental designs may provide a more comprehensive understanding of how AI adoption develops in educational settings.

Finally, this study focuses on the core constructs of the UTAUT2 framework. While this provides a structured analytical lens, incorporating additional variables—such as learning motivation, self-regulation, or technology-related anxiety—may offer a more comprehensive explanation of students' engagement with AI tools.

## Data Availability

The datasets generated and analyzed during the current study are not publicly available due to restrictions imposed by the ethics committee to protect participant confidentiality. However, anonymized data may be made available by the corresponding author upon reasonable request and with appropriate institutional approval. Requests for access to the datasets should be directed to xusharon6@gmail.com.
