# Supplementary material for: Applying UTAUT2 model to investigate acceptance of DeepSeek by Chinese university students for academic use
Source: Front Psychol. 2026 May 15;17:1828331. doi: 10.3389/fpsyg.2026.1828331 (PMC13220166; doi:10.3389/fpsyg.2026.1828331)
Supplement: Supplementary file 1 [file Supplementary_file_1.docx]

Appendix. Measurement Items

This study employed a structured questionnaire to measure all constructs. All items were adapted from prior UTAUT2-based studies and modified to fit the context of DeepSeek use in academic learning. Unless otherwise specified, all items were measured using a seven-point Likert scale ranging from 1 = strongly disagree to 7 = strongly agree.

A1. Demographic Information

Gender: Male / Female

Age: Under 19 / 20–22 / 23–25 / Above 26

A2. Performance Expectancy (PE)

PE1: I find DeepSeek useful for my learning.

PE2: Using DeepSeek increases my chances of achieving things that are important to me.

PE3: Using DeepSeek helps me accomplish learning-related tasks more quickly.

PE4: Using DeepSeek improves my learning efficiency.

A3. Effort Expectancy (EE)

EE1: Learning how to use DeepSeek is easy for me.

EE2: My interaction with DeepSeek is clear and understandable.

EE3: I find DeepSeek easy to use.

EE4: It is easy for me to become skillful at using DeepSeek.

A4. Social Influence (SI)

SI1: People who are important to me think that I should use DeepSeek in my learning.

SI2: People who influence my behavior think that I should use DeepSeek.

SI3: People whose opinions I value prefer that I use DeepSeek in my learning.

A5. Facilitating Conditions (FC)

FC1: I have the resources necessary to use DeepSeek in my learning.

FC2: I have the knowledge necessary to use DeepSeek.

FC3: DeepSeek is compatible with other technologies I use.

FC4: I can get help from others when I have difficulties using DeepSeek.

A6. Hedonic Motivation (HM)

HM1: Using DeepSeek in my learning is fun.

HM2: Using DeepSeek is enjoyable.

HM3: Using DeepSeek in learning is exciting.

A7. Price Value (PV)

PV1: DeepSeek is reasonably priced.

PV2: The benefits of using DeepSeek are worth the cost.

PV3: DeepSeek provides good value for money.

A8. Habit (HT)

HT1: Using DeepSeek has become a habit for me.

HT2: I am addicted to using DeepSeek.

HT3: I feel the need to use DeepSeek.

HT4: Using DeepSeek has become natural to me.

A9. Behavioral Intention (BI)

BI1: I intend to continue using DeepSeek in my learning in the future.

BI2: I will always try to use DeepSeek in my learning.

BI3: I plan to use DeepSeek frequently in my learning.

A10. Use Behavior (UB)

UB1: I frequently use DeepSeek in my learning.

UB2: Using DeepSeek is a pleasant experience.

UB3: I currently use DeepSeek as a learning support tool.

UB4: I spend a considerable amount of time using DeepSeek in my learning.
